# MicrobeAnnotator: a user-friendly, comprehensive functional annotation pipeline for microbial genomes

**DOI:** 10.1186/s12859-020-03940-5

**Published:** 2021-01-06

**Authors:** Carlos A. Ruiz-Perez, Roth E. Conrad, Konstantinos T. Konstantinidis

**Affiliations:** 1 School of Biological Sciences, Georgia Institute of Technology, Atlanta, GA 30332 USA; 2 Ocean Science and Engineering, School of Biological Sciences, Georgia Institute of Technology, Atlanta, GA 30332 USA; 3 School of Civil and Environmental Engineering, Georgia Institute of Technology, Atlanta, GA 30332 USA; 4 Center for Bioinformatics and Computational Genomics, Georgia Institute of Technology, Atlanta, GA 30332 USA

**Keywords:** Genome annotation, Comparative genomics, Protein annotation, Metabolic potential

## Abstract

**Background:**

High-throughput sequencing has increased the number of available microbial genomes recovered from isolates, single cells, and metagenomes. Accordingly, fast and comprehensive functional gene annotation pipelines are needed to analyze and compare these genomes. Although several approaches exist for genome annotation, these are typically not designed for easy incorporation into analysis pipelines, do not combine results from different annotation databases or offer easy-to-use summaries of metabolic reconstructions, and typically require large amounts of computing power for high-throughput analysis not available to the average user.

**Results:**

Here, we introduce MicrobeAnnotator, a fully automated, easy-to-use pipeline for the comprehensive functional annotation of microbial genomes that combines results from several reference protein databases and returns the matching annotations together with key metadata such as the interlinked identifiers of matching reference proteins from multiple databases [KEGG Orthology (KO), Enzyme Commission (E.C.), Gene Ontology (GO), Pfam, and InterPro]. Further, the functional annotations are summarized into Kyoto Encyclopedia of Genes and Genomes (KEGG) modules as part of a graphical output (heatmap) that allows the user to quickly detect differences among (multiple) query genomes and cluster the genomes based on their metabolic similarity. MicrobeAnnotator is implemented in Python 3 and is freely available under an open-source Artistic License 2.0 from https://github.com/cruizperez/MicrobeAnnotator.

**Conclusions:**

We demonstrated the capabilities of MicrobeAnnotator by annotating 100 *Escherichia coli* and 78 environmental Candidate Phyla Radiation (CPR) bacterial genomes and comparing the results to those of other popular tools. We showed that the use of multiple annotation databases allows MicrobeAnnotator to recover more annotations per genome compared to faster tools that use reduced databases and is computationally efficient for use in personal computers. The output of MicrobeAnnotator can be easily incorporated into other analysis pipelines while the results of other annotation tools can be seemingly incorporated into MicrobeAnnotator to generate summary plots.

## Background

The recovery of microbial genomes from different environmental, clinical, and industrial samples has exponentially increased over the last decade thanks to high-throughput sequencing, with > 100,000 genomes currently available [1]. These complete or partial genomes, in the form of isolate genomes, single-cell amplified genomes (SAGs), or metagenome-assembled genomes (MAGs), can provide not only taxonomic information about the composition of the microbial community but can also offer valuable information about the metabolic roles that members of the community potentially play [2–4]. A crucial step in assessing the metabolic role of community members is the functional gene prediction and metabolic reconstruction [5]. The functional gene data can also be used to infer the metabolic differences with close relatives, characterize novel potential metabolisms, or detect the presence of antibiotic resistance genes or toxins, among other genes of interest [6, 7].

The functional annotation starts with gene identification or gene calling (structural annotation), which can be automatically performed using several tools, including (Meta)Prodigal (PROkaryotic DYnamic programming Gene-finding ALgorithm), (Meta)GeneMark, and MetaGeneAnnotator, among others [8–10]. The next annotation step relies on the use of reference protein databases to assign functions to query (unknown) protein sequences based on homology or orthology searches (functional annotation) [11–13]. Some of the databases used are comprehensive, including protein sequences derived from complete and partial genomes and are usually updated periodically; widely used examples include the Universal Protein Resource (UniProt) [14] and Reference Sequence Database (RefSeq) [15], Integrative Protein Signature Database (InterPro) [16], Protein Families Database (Pfam) [17] and the database and infrastructure for comparative genomics (SEED) [18] databases. On the other hand, specialized databases aim at curating the entries to include only protein sequences belonging to specific functions or protein families of interest, e.g., The Comprehensive Antibiotic Resistance Database (CARD) for antibiotic resistance proteins [19]. The methods used to search query protein sequences against these databases vary in complexity, comprehensiveness, speed, scalability, and results. For example, the National Center for Biotechnology Information (NCBI) Prokaryotic Genome Annotation Pipeline (PGAP, [20]), Prokka (prokaryotic annotation) [21], RAST (Rapid Annotations using Subsystem Technology) [22], and DRAM (Distilled and Refined Annotation of Metabolism) [23] start from genomes and predict genes and proteins, tRNAs, rRNAs, and perform functional annotation of the predicted proteins. Others such as InterProScan [12] and EggNOG-Mapper (the evolutionary genealogy of genes: Non-supervised Orthologous Groups) [11] start from already predicted protein sequences and perform functional annotations using mostly Blast (Basic Local Alignment Search Tool) [24], Diamond (double index alignment of next-generation sequencing data) [25], or HMMER [26] as search tools.

Besides the initial input data, annotation tools may also differ in the reference databases used, the extent (level) of annotation provided (e.g., individual gene vs. pathway level), and result outputs. For instance, tools such as Prokka and RAST that are fast often rely on smaller, curated databases to speed-up searching times while maintaining high-quality annotations. Although these tools offer fast run times and high-quality annotations, they can leave several proteins unannotated, especially in more divergent or novel genomes. On the other hand, more complex genome annotation tools such as DRAM use several databases to return comprehensive annotations at the expense of increased computational resources and time. In any case, one unifying theme among most annotation tools is that the text-based outputs they return to the user have to be parsed to make further sense of the data. While web-based tools such as the KEGG Mapper tools [27] and MAPLE (Metabolic And Physiological potentiaL Evaluator, now GenoMAPLE) [28] provide graphical outputs that are user-friendly and easy to parse, they lack in scalability, especially when dealing with more than a couple of genomes.

Given the strengths and weaknesses of existing tools, it is evident that there is a need for an annotation tool that is easy to use, comprehensive, high throughput while using less computational resources than complex tools and providing summaries of annotation results that users with little programming experience can take advantage of. To fill this gap, we present MicrobeAnnotator, a python-based command-line tool that employs multiple reference databases for the automated functional annotation, summarization, and comparison of microbial genomes. MicrobeAnnotator can use multiple processing cores to annotate several genomes simultaneously or speed up individual genome annotation.

## Implementation and outputs

### MicrobeAnnotator database building

There are two main running modes for MicrobeAnnotator, *standard* and *light*. The *standard* mode uses the KOfam [29], UniProt’s Swissprot and trEMBL [30], and NCBI’s RefSeq [31] databases, while the *light* mode uses only the first two. Before running MicrobeAnnotator, the databases used by the program must be downloaded and formatted. To do this, the user has to execute the *microbeannotator_db_builder* script, which allows the selection of output folders for the databases, the program intended for searching, i.e., Blast [24], Diamond [25], or Sword (Smith–Waterman on Reduced Database) [32], and the number of threads to use. This script runs in five main steps: (1) Download the databases (protein sequences and metadata); (2) Parse annotation metadata associated with the downloaded protein sequences; (3) Build the SQLite databases with annotation data, including accessions to other databases (e.g. KO, E.C., GO, Pfam, and InterPro); (4) Build databases for the interconversion between KO–E.C. and InterPro–E.C. identifiers; and finally, (5) Build the databases or files required for the homology search method selected by the user. If the program fails at any of these stages for any reason, the users can resume the script by selecting the step they want to restart.

### MicrobeAnnotator functional annotation process

**Fig. 1:**
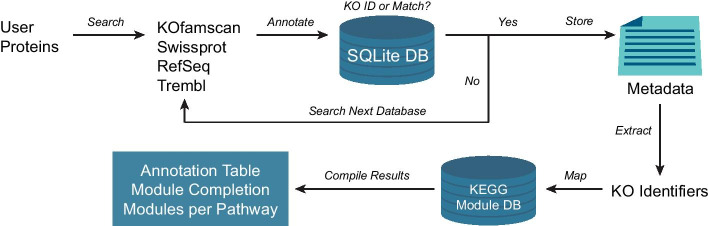
Graphical summary of the MicrobeAnnotator pipeline. Starting from a set of protein sequences, MicrobeAnnotator iteratively searches against 2 or 4 databases depending on the mode used (*standard* or *light*). Proteins without a KO identifier or match are searched against the next database. Otherwise, its metadata (best match, product, KO identifier, the taxonomy of best hit, GO numbers, and Pfam and InterPro accessions) are stored. Finally, KO identifiers are extracted, and module completeness is calculated using the custom MicrobeAnnotator database. The results are compiled in a single matrix-like module completeness table and summary plots for all genomes combined

Figure 1 shows a graphical representation of the MicrobeAnnotator functional annotation pipeline. Considering the variety of tools and parameters available for gene prediction [8–10], MicrobeAnnotator does not predict proteins from contings or genomes but instead expects predicted protein sequences in FASTA format as input. The user can provide one or multiple files (for multiple genomes) that are serially or simultaneously annotated depending on the number of cores available. The functional annotation pipeline consists of four main steps for each file of protein sequences provided (Fig. 1). The first three involve searches against the databases, extraction of best matches, and linking best matches to annotations in MicrobeAnnotator’s custom SQLite database, while the final step compiles all the information extracted and summarizes the data. Given the large size of the databases used by MicrobeAnnotator, we have implemented an iterative annotation pipeline that takes advantage of each database curation level and size to speed up the process while providing the most reliable annotations. The detailed steps of the pipeline are:

1. All proteins are searched against the curated KEGG Ortholog (KO) database using KOfamscan [29, 33]; best matches are selected according to Kofamscan’s adaptive score threshold. The annotation and KO identifier for each match are saved.
2. Proteins without KO identifiers (or matches) are extracted and searched against Swissprot (using the selected search tool). Filtering parameters for a match can be modified; otherwise, the defaults are used (i.e., 40% amino-acid identity, bitscore 80, and alignment length 70%). The annotation and KO identifiers for each match are saved.
3. Proteins without a KO identifier (or match in Swissprot) are extracted and searched against the curated RefSeq database. Annotation and KO identifiers for each match are saved. This means that if the tool finds a match without a KO identifier in Swissprot, the protein is still searched against RefSeq.
4. Proteins without a KO identifier (or match in RefSeq) are extracted and searched against the non-curated trEMBL database. Annotation and KO identifiers for each match are saved.
5. All protein annotations are compiled in a single table per genome, which includes all metadata associated with each best match. This table may contain more than one annotation per protein, depending on the assignment of a KO identifier associated with the protein during steps 1 through 4 above. The KO identifiers associated with all proteins in each genome (or set of proteins) are extracted, and KEGG module completeness is calculated based on the total steps in a module, the proteins (KOs) required for each step, and the KOs present in each genome. KEGG modules are defined as functional gene units that are linked to higher metabolic capabilities (pathways), structural complexes, and phenotypic characteristics. For example, module M00001 (Glycolysis, Embden-Meyerhof pathway; glucose =  > pyruvate) is part of the Glycolysis/Gluconeogenesis pathway (00010). Module completeness is then summarized in a matrix with all genomes included in the analysis.

To provide greater flexibility to the user, MicrobeAnnotator supports the use of three different popular search tools, i.e., Blast, Diamond, and Sword. The user can also modify the filtering thresholds used to select the best match found in the databases (i.e., percent identity, bitscore, e-value, or percent alignment across the matching reference protein sequence) and to select whether clustering for the graphical output should be performed or not. Finally, multiprocessing is also supported with the -t (threads) and -p (processes) options. The processes option determines the number of input files (genomes) that are simultaneously processed, while the threads option determines the number of computing cores used per file being processed. For instance, if the user has two genomes and selects -p 2 and -t 4, MicrobeAnnotator will use 8 cores in total for the annotation, 4 for each genome that will be processed simultaneously.

### MicrobeAnnotator output

MicrobeAnnotator produces a single main output folder per run; if the run contains multiple genomes, all the information for each genome will be saved within the main output folder. This folder, designated by the user, includes an “*annotation_results*” folder that contains the compiled annotations and search results for each genome, as shown in Table 1. The folder includes a file with the annotations and metadata for each protein and a file with only the KO identifiers recovered per genome. Individual folders with the raw and filtered best matches per database are also created, i.e., one folder with KOfamscan, Swissprot, RefSeq, and trEMBL results. Finally, the summarized results include a matrix file with KEGG module completeness for all genomes, a heatmap summarizing the completeness of modules that are above 50% completes in at least one genome, and a bar plot showing the number of modules above 80% completeness grouped by the pathway that the modules are linked to. All these files are explained in greater detail in the supplementary online material.

**Table 1:** Folders and files produced by MicrobeAnnotator

Result (type)	Description of contents
Annotation_results (folder)	Annotations and KO numbers per genome
Kofamscan_results (folder)	Raw and filtered KOfamscan results
Swissprot_results (folder)	Raw and filtered Swissprot results
Refseq_results (folder)	Raw and filtered RefSeq results
Trembl_results (folder)	Raw and filtered trEMBL results
[prefix].tab (file)	Global table with annotations
[prefix]_heatmap.pdf (file)	Module completeness heatmap
[prefix]_barplot.pdf (file)	Barplot of modules above 80% complete

## Results

### Computing Requirements of MicrobeAnnotator compared to other tools

**Fig. 2:**
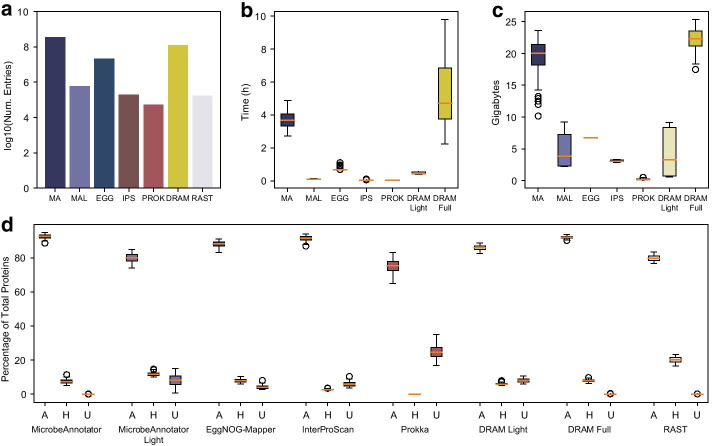
Annotation tool comparison using *E. coli* genomes. The number of entries in the databases (**a**) used by the different annotation tools showed that MicrobeAnnotator has the highest number of database entries compared to other tools. The annotation of 100 *E. coli* genomes showed that MicrobeAnnotator and DRAM, being the most comprehensive tools, required more than 2 h to annotate each genome using 10 threads (**b**). Faster tools such as Prokka compared with the *light* version of MicrobeAnnotator, requiring only minutes to perform the annotations. The more extensive databases used by MicrobeAnnotator resulted in higher RAM usage in par to that required by DRAM (**c**). Finally, the percentage of proteins classified as *annotated* (*A*), *hypothetical* (*H*), and *unannotated* (*U*) showed that MicrobeAnnotator could annotate more than 90% of proteins for well-represented genomes. In comparison, the *light* version still annotates ~ 80% of the proteins, suggesting it is an efficient annotation option for microorganisms with good representation in the databases (**d**). *MA* MicrobeAnnotator, *MAL* MicrobeAnnotator Light, *EGG* EggNOG-Mapper, *IPS* InterProScan, *PROK* Prokka

We compared MicrobeAnnotator to other popular genome annotation pipelines, including Prokka v1.14.6 [8], RAST [7], EggNOG-mapper v2.0.1b-4-g4c2b55e, InterProScan v5.47-82.0 and DRAM. The first comparison was in terms of database entries used by each tool. As previously suggested [23], we counted the number of FASTA protein entries, HMM models, or website database size reports (for RAST and InterProScan), depending on the tool. Figure 2a shows that MicrobeAnnotator and DRAM have orders of magnitude more entries than the other tools, and MicrobeAnnotator (~ 350 million) has almost three times the number of entries compared to DRAM (~ 121 million). This difference is mostly driven by the inclusion of NCBI’s RefSeq database and UniProt trEMBL compared to the UniRef90 used in DRAM. While most of the additional sequences used by MicrobeAnnotator are redundant among themselves and/or with sequences in UniRef90, a few are not redundant (e.g., recently published/determined annotations); thus, they represent a more comprehensive database for annotation. Further, the additional (redundant) sequences may be important for more precise taxonomic identification of the best match, representing useful information for many users.

More important than the number of entries per tool, we compared each tool's annotation performance in terms of speed, memory usage, proteins annotated, and level of consistency between tools. For this, we annotated 100 *E. coli* genomes selected, at random, from the latest NCBI RefSeq genome release (Additional file 2: Table S1). For this analysis, we included both modes of MicrobeAnnotator (*standard* and *light*). Likewise, considering the high RAM requirements of DRAM, we performed two annotations with [DRAM full] and without [DRAM light] the UniRef90 database. Given that MicrobeAnnotator offers flexibility in searching tools, we first determined the optimal search options to annotate these genomes. Based on the results shown in the Supplementary online section and Additional file 1: Figures S2 and S3, Sword gave the best compromise between speed and sensitivity; thus, we used Sword for the remaining analyses. Finally, given that not all tools allow the annotation of multiple genomes in a single run, each genome was annotated independently using 10 threads per run.

Of the tools tested, InterProScan and Prokka were the fastest, taking on average 2.7 and 2.5 min per genome, respectively (Fig. 2b). These were closely followed by the *light* version of MicrobeAnnotator that took on average 6.4 min to annotate a typical *E. coli* genome. The *standard* mode of MicrobeAnnotator required significantly less time to fully annotate a genome (3,7 h) compared to DRAM when using the UniRef90 database (~ 5.1 h; Fig. 2b), even though MicrobeAnnotator has three times the number of entries compared to DRAM (discussed above). If a faster annotation is desired, MicrobeAnnotator (*standard* mode) using Diamond as the search tool can annotate a single genome in approximately 26 min, with a minor loss in sensitivity related to distant matches in the databases (Additional file 1: Figure S2). Accordingly, the use of the *light* mode in MicrobeAnnotator with Diamond takes even less time, being closer to the fastest tool, Prokka. We also tested the *annotate_genes* mode of DRAM that uses proteins as input and only performs functional annotation and obtained similar results in terms of annotation times (data not shown). Finally, in our tests and depending on the server load, the web version of RASTtk can take up to 120 min (this includes queuing and processing time) while the local version takes approximately 20 min per genome. Considering the large memory requirements of DRAM, which could make it impractical for individual computers [23], we also compared the RAM usage by each tool (Fig. 2c). In this test, Prokka had the lowest RAM requirement with ~ 204 MB per genome. Not surprisingly, MicrobeAnnotator (*standard* mode) and DRAM (full) required on average 19.4 and 22.1 GB of RAM per genome; this difference in RAM requirements was statistically significant (Mann–Whitney *p-adj* < 0.05). Finally, the *light* version of MicrobeAnnotator had a RAM usage of 4.7 GB on average, close to DRAM without the use of UniRef90 (4.2 GB), indicating that both “modes” of the tools are suitable for more resource-limited computing environments. In addition to the RAM required at runtime, DRAM requires large amounts of RAM to build the necessary databases; according to the README file, it requires 512 GB of RAM to build all databases if the UniRef90 is included, and at least 64 GB if it is skipped. It is important to recognize, however, that DRAM was designed for high-performance computing clusters with considerable computing resources, whereas MicrobeAnnotator is intended for a more general-purpose usage under more limited computational resources.

### Annotation quality of MicrobeAnnotator compared to other tools

We compared the annotation results for all 100 *E. coli* genomes obtained with MicrobeAnnotator and the other tools in terms of proteins annotated, how consistent or similar these annotations were to one another, and the summarization capabilities of each tool. In this regard, we followed DRAM’s classification scheme [23], where the annotation classification of each protein was slightly different for each tool. For MicrobeAnnotator, a protein was classified as *annotated* if it had at least one match in KOfamscan, Swissprot, RefSeq, or trEMBL, and the annotation did not contain ‘*hypothetical*,’ ‘*uncharacterized*,’ ‘*domain of unknown function*,’ or ‘*protein of unknown function*.’ A *hypothetical* classification was assigned when the protein had a match, but it contained any of the above terms. An *unannotated* protein was assigned when no match against any database was found (identified with ‘*No match found*’ in MicrobeAnnotator’s output). This classification scheme was the same for DRAM and InterProScan. In the case of eggNOG-mapper, Prokka, and RAST, the classification was slightly different. A protein was *annotated* when a match was found, and the annotation did not contain any hypothetical terms. For eggNOG-mapper, a *hypothetical* protein had an annotation with any of the terms above (or no protein description), while an *unannotated* protein did not appear in the output. In the case of RAST and Prokka, it is difficult to discriminate between conserved hypothetical proteins and no matches found in the database in the output. Thus, for RAST, a *hypothetical* protein had an annotation containing any of the terms above, and if the annotation contained *“hypothetical protein,”* it should be accompanied by additional information such as one FIGFAM entry associated to be considered *hypothetical*; otherwise, it was considered *unannotated*. For Prokka, where the differentiation between hypothetical and unannotated was impossible, we followed the classification established in [23], where all *hypothetical* proteins were considered *unannotated*.

In general, most tools could annotate 80% or more proteins of the *E. coli* genomes, except for Prokka and RAST, which annotated ~ 75% and ~ 79.7%, on average (Fig. 2d). Prokka had the highest percentage of unannotated proteins (~ 24.6%), while RAST had the highest percentage of hypothetical proteins (~ 24.7%). Overall, MicrobeAnnotator, DRAM (using UniRef90), and InterProScan annotated the most proteins compared to all other tools (~ 92.5%, ~ 92.06%, and ~ 91,3%, respectively). Like DRAM, MicrobeAnnotator had the lowest number of unannotated proteins (~ 0.08%), indicating that the use of several comprehensive databases allows for the discovery of conserved hypothetical proteins (or other functions) that could have potential biological importance [34, 35]. Therefore, even if MicrobeAnnotator run times are longer than those for EggNOG-Mapper, InterProScan, and Prokka, the resulting annotations have more annotated proteins (Fig. 2). Moreover, if the Diamond option in MicrobeAnnotator is used, the runtimes can be reduced from hours to minutes, without a noticeable loss in annotation performance, revealing that this option is a more competitive annotation compared to DRAM.

To test how comprehensive and consistent the annotations of each tool are, we evaluated the presence/absence of identifiers in the output, such as E.C. numbers. Out of all the tools tested, RAST had the lowest number of identifiers, including FigFam identifiers in ~ 2.1% of the annotations and E.C. numbers in ~ 25.8% of the annotations. Prokka annotations, on the other hand, included E.C. numbers in ~ 34.1% of the annotations and Cluster of Orthologous Groups (COG) identifiers in ~ 60.1% of the cases. The remaining tools (MicrobeAnnotator, InterProScan, EggNOG-Mapper, and DRAM) consistently included several additional identifiers from multiple databases to complement the text-based annotation descriptions. The most prevalent of these identifiers were KEGG KO identifiers, which MicrobeAnnotator and DRAM use to summarize metabolic potential into pathways and modules. Therefore, to compare each tool's breadth of annotations, we extracted all KO identifiers from each output and used them as input to MicrobeAnnotator to create summary statistics. This was possible thanks to the additional scripts available as part of the MicrobeAnnotator pipeline that allow users to import KO identifiers and create summaries. For tools that included E.C. numbers in their output, we also developed an additional script to translate KO identifiers to E.C. numbers and *vice versa*; this script is also available as part of MicrobeAnnotator’s pipeline. Unfortunately, although InterProScan provides several protein identifiers from other databases, e.g., The Institute for Genomic Research's database of protein families (TIGRFAM), and protein analysis through evolutionary relationships database (PANTHER), it does not provide any KO or E.C. identifiers for comparisons.

The summary of this analysis for each tool can be found in Additional file 2: Tables S3–S9; a graphical representation of the annotation summary from MicrobeAnnotator is also available in Additional file 1: Figure S4. As expected, the metabolic potential recovered from all tools and summarized for all *E. coli* genomes showed similarities in carbon, vitamin, amino acid, and fatty acids metabolism functions, possibly reflecting that these functions are part of the *E. coli* core genome [14]. However, several key differences in aromatics metabolism and degradation of secondary metabolism compounds can be easily identified among the genomes from the heatmap based on all tools. For instance, the *E. coli* genomes could be separated and clustered together in groups that reflect the presence of pathogenicity signals (toxins and secretion systems) (Additional file 1: Figure S6), consistent with previous classification schemes [15]. The total number of modules per pathway shown in the bar plots generated by MicrobeAnnotator can also serve to identify incomplete genomes or missing complete pathways. This information, combined with protein marker completeness estimations, can give the user information about pathways truly missing from the genome vs. being assembly artifacts or sequencing gaps.

To compare how similar the resulting annotation matrices from different tools are to one another, we computed the Frobenius norm of the difference between a pair of matrices as follows:

$$d_{2} \left( {A,B} \right) = \sqrt {\mathop \sum \limits_{i = 1}^{m} \mathop \sum \limits_{j = 1}^{n} \left( {a_{ij} - b_{ij} } \right)^{2} }$$

where *A* and *B* are the two matrices to be compared, *m* and *n* are the number of rows and columns in the matrices, *a* is an element in the *i*th and *j*th position in matrix *A*, and *b* is an element in the *i*th and* j*th position in matrix *B*. For this calculation, the matrices must have the same order. Two *E. coli* genomes were not annotated by RAST, and thus, were removed from all matrices to maintain the same dimensions.

The MDS ordination of the values obtained using the Frobenius norm showed that both modes of MicrobeAnnotator (*standard* and *light*) were similar and adjacent to the two DRAM modes (Additional file 1: Figure S7). This indicated that the *light* mode of MicrobeAnnotator can recover similar metabolic reconstructions to those obtained using MicrobeAnnotator’s *standard* mode and DRAM’s full mode. Considering the differences in run times between the *standard* and *light* versions of MicrobeAnnotator (Fig. 2) and depending on the query genome and the representation of close relatives on the databases, the *light* mode of MicrobeAnnotator can provide robust, comprehensive annotations for microbial genomes. In the case of *E. coli*, 99,5% of the modules were identified with the same completeness level using both MicrobeAnnotator modes. Moreover, the EggNOG-Mapper annotations were closer to MicrobeAnnotator and DRAM annotations, while RAST and Prokka were the most distinct. Upon closer inspection, this result is due to differences in identifying proteins involved in several metabolic pathways (Additional file 2: Table S10). For instance, out of 394 modules summarized, MicrobeAnnotator (and DRAM) detected 26 and 23 modules that were at least 20% more complete than those summarized using Prokka and RAST, respectively. These modules mostly belonged to carbohydrate metabolism pathways, ATP synthesis, and drug resistance. In contrast, Prokka detected 101 modules that were at least 20% more complete than those detected by MicrobeAnnotator and DRAM, while RAST detected 75 such modules. These modules were linked with varied metabolism types, including aromatics degradation, biosynthesis of secondary metabolites, drug resistance, glycan, and lipid metabolism. Nonetheless, ~ 47% of these modules were below 50% complete, suggesting that the annotation matches may not be strong (e.g., distant matches), and probably require closer inspection. Moreover, it is important to note that a single E.C. identifier may be linked to multiple KO identifiers indicating that the original protein might not be a real functional ortholog of the original KO record. For instance, E.C. 2.7.7.-, which was present in almost all RAST and Prokka annotations, includes a group of ~ 100 types of adenylyl transferases and has links with 17 KO identifiers. These one-to-many links exhibited by E.C. numbers accounted for the seemingly better recovery of metabolic modules found for Prokka and RAST, the only two tools that required translation of E.C. to KO identifiers. A quick test using the original MicrobeAnnotator summaries complemented with translated E.C. identifiers (also extracted from the original MicrobeAnnotator annotations) showed a similar pattern to that observed for Prokka and RAST (Additional file 2: Tables S10–S11). In this case, the “complemented” version of the original MicrobeAnnotator summary found 37 modules that were 20% more complete than the original summary. This result urges caution when importing identifiers from other annotation tools, and therefore we recommend the use of this feature within MicrobeAnnotator only for manually verifying results and exploratory purposes.

### Annotation of poorly represented (or studied) genomes

**Fig. 3:**
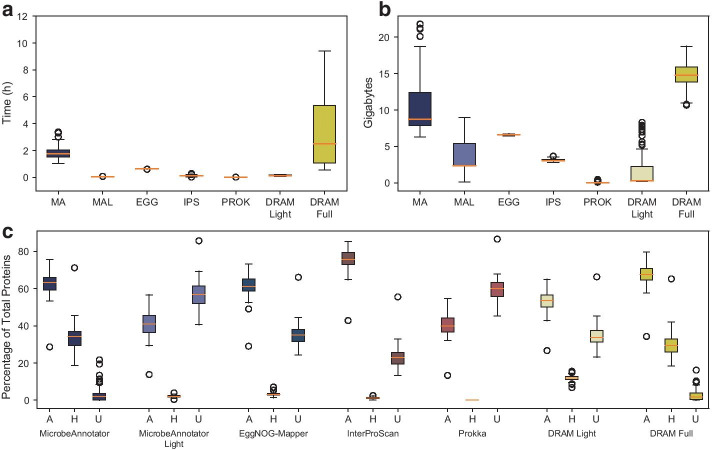
Annotation tool comparison using CPR bacteria genomes. Given the smaller genome sizes and lower completeness levels, all tools required shorter times to perform annotations (**a**). While the *standard* mode of MicrobeAnnotator only takes ~ 2 h to annotate a genome, the *light* version takes only a couple of minutes like other tools that use smaller databases. Consequently, the RAM usage for these smaller genomes decreases but is still on par with DRAM requirements (**b**). As expected for genomes with lower representation in the databases, all tools could annotate a lower percentage of proteins. However, MicrobeAnnotator was able to find more matches for hypothetical proteins, having one of the lowest percentages of unannotated proteins of all tools (**c**). *MA* MicrobeAnnotator, *MAL* MicrobeAnnotator Light, *EGG* EggNOG-Mapper, *IPS* InterProScan, *PROK* Prokka. A: Annotated, H: Hypothetical, U: Unannotated

We demonstrated above that MicrobeAnnotator could annotate most proteins in *E. coli* genomes and recover similar summaries compared to more complex and computationally demanding tools such as DRAM, while having better annotation calls compared to less demanding tools such as Prokka and RAST. Nonetheless, the *E. coli* genomes are amongst the most widely studied, sequenced, and reported genomes, making them relatively easy to annotate for any tool. To evaluate MicrobeAnnotator against higher genomic novelty, we decided to annotate a group of 78 genomes from the Candidate Phyla Radiation (CPR) group. These genomes were recently described and have unusual metabolic capacity with an increased number of hypothetical or poorly characterized proteins compared to *E. coli* [36, 37]. One additional unifying feature of these genomes is that most of them were recovered from SAGs or MAGs, providing a useful test dataset in terms of genome completeness. Our comparisons showed that there is a similar pattern in terms of computing time with the *E. coli* dataset mentioned above, with MicrobeAnnotator and DRAM (with UniRef90) requiring longer times to complete the annotations (~ 1.8 h and ~ 3.2 h per genome, respectively), followed by EggNOG-Mapper (~ 1.1 h; Fig. 3a). The remaining tools were quite fast at annotating these genomes, requiring less than 10 min in all cases. All annotation times were lower compared to the *E. coli* annotations, mainly due to the smaller number of proteins to be annotated, i.e., an average of 794 in CPR vs. 4,600 proteins in *E. coli* genomes.

Regarding the percentage of proteins annotated, the tool with the highest percentage of annotated proteins was InterProScan with an average of 75.5%, followed by DRAM (using UniRef90) with ~ 67.6% and MicrobeAnnotator with ~ 62.9% (Fig. 3). On average, 34% of the proteins that were not annotated by MicrobeAnnotator but were annotated by InterProScan contained the word “domain” or “consensus disorder prediction;” that is, they represented only general function predictions. Based on our definition of annotated, hypothetical, and unannotated proteins, these instances would be classified as annotated, but it is difficult to derive function from domains and profile predictions, which often occur in InterProScan. Therefore, we expect that the differences between the percentage of proteins annotated with specific functions using MicrobeAnnotator (and DRAM) and InterProScan to be smaller than the above numbers indicated. Interestingly, although InterProScan annotated a higher percentage, it also had a higher percentage of proteins that remain unannotated (~ 23.1%), compared with MicrobeAnnotator (~ 3.1%) and DRAM (~ 2.6%). These unannotated proteins from InterProScan were classified as hypothetical in most cases by MicrobeAnnotator (more than 80% of cases). Therefore, although no annotation was recovered from MicrobeAnnotator, there is value in knowing the protein was found in the databases (in other genomes) as a hypothetical conserved protein.

**Fig. 4:**
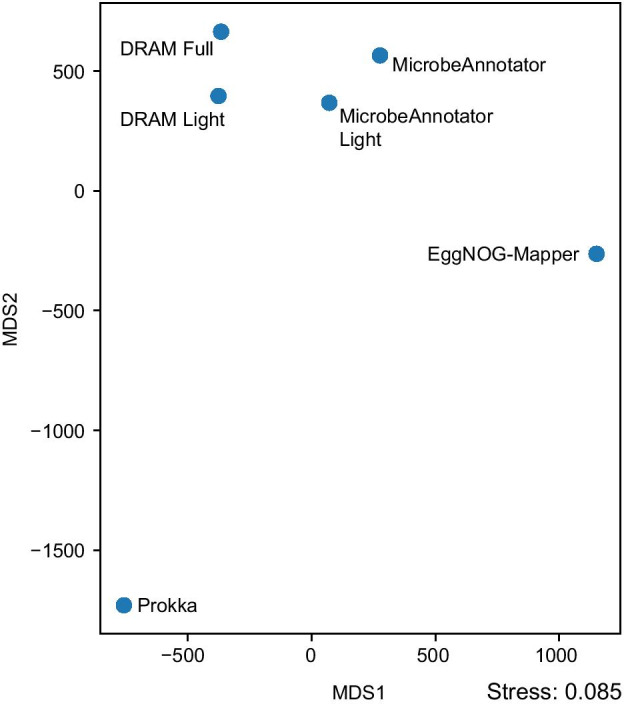
Multi-dimensional scaling ordination of annotation matrix distances. The distance between annotation summary matrices for CPR genomes showed that both modes of MicrobeAnnotator recover similar KO-based summaries that, at the same time, are similar to those obtained using DRAM. Prokka and EggNOG-Mapper recovered different modules highlighting the advantage of using multiple annotation tools and incorporating their results into MicrobeAnnotator

The metabolic summary of CPR genomes obtained using MicrobeAnnotator revealed substantial differences compared to the *E. coli* genomes. The first evident characteristic is the large fraction of largely incomplete or absent metabolic modules in CPR genomes (Additional file 1: Figure S8). While most CPR genomes showed the metabolic potential for primary metabolism, with the capacity to carry out nucleotide biosynthesis, glycolysis, the Calvin cycle, and the pentose phosphate cycle (Additional file 1: Figure S8, bottom), a reduced number encode the capacity to perform isoprenoid biosynthesis (mevalonate and non-mevalonate pathways), which has been previously reported for members of the CPR [38]. Further, the high level of incompleteness of these genomes prevented several modules from being identified at a larger scale using any tool; hence, the gene-by-gene annotations obtained by MicrobeAnnotator and other tools become essential to curate and identify potential metabolic capabilities manually. In this regard, the summary matrix distance of all tools showed similar patterns to those obtained previously for the *E. coli* dataset. First, consistent with previous results, both modes of MicrobeAnnotator were closer together, where 100% of the modules found were within 10% difference in completeness. Both modes of DRAM were also closer to MicrobeAnnotator results (Fig. 4). This was expected because most of the KO identifiers come from the KEGG database that both tools use, with the (small) differences observed likely originated from the use of RefSeq/UniProt and UniRef90 for MicrobeAnnotator and DRAM, respectively. Consistent with previous results, Prokka appeared as the most different tool (Fig. 4), with the interesting finding of additional antibiotic resistance modules, including cationic antimicrobial and imipenem resistance (Additional file 2: Tables S12–S13). Again, the use of additional identifiers from other databases could be useful and complementary but should be inspected carefully to avoid false positives.

## Conclusions

Annotating and comparing microbial genomes is currently performed using different bioinformatics tools and approaches with different advantages and requirements. On several occasions, however, these tools require users to manually parse through text-based results to obtain meaningful information. Further, most users lack the computational capabilities to compare multiple genomes at the same time. In addition, fast annotation tools often use smaller databases, resulting in less comprehensive annotations, especially for underrepresented organisms in the databases. MicrobeAnnotator effectively addressed these limitations by using multiple databases in an iterative fashion, which allows it to balance the tradeoffs of using large databases while providing the user with comprehensive annotations and easy-to-understand results that can be used to guide the next, more detailed analyses. Compared to more complex annotation tools, MicrobeAnnotator has lower computational demands and, depending on the options used, can be competitive with faster tools that use smaller databases. The examples highlighted above underscore the capabilities of MicrobeAnnotator for quickly annotating and comparing groups of genomes of well-studied or not-well-studied microbial taxa. While MicrobeAnnotator is a standalone program, its results can be seemingly incorporated into other analysis pipelines. The results obtained here also highlighted the importance of using different annotation approaches that offer complementary results. In this regard, MicrobeAnnotator allows users to import results from other annotation pipelines to create combined summaries and provide the translation of identifiers from other databases to the ones used in MicrobeAnnotator, increasing its flexibility and probable use case. Hence, we anticipate that MicrobeAnnotator will find many applications in microbiome and genome research across clinical or environmental settings.

## Availability and requirements

*Project name* MicrobeAnnotator

*Project home page* https://github.com/cruizperez/MicrobeAnnotator

*Operating system(s)* Platform independent

*Programming language* Python

*Other requirements* IBM Aspera Connect

*License* Artistic License 2.0

*Any restrictions to use by non-academics* Those stated in Artistic License 2.0

## Supplementary Information

**Additional file 1.** Supplementary results and figures.

**Additional file 2.** Tables with genomes used in this study and annotation result summaries for each tool tested.

## Data Availability

Data generated or analyzed during this study are included in this published article or as supplemental material. Additional data can be obtained from the authors upon request.

## Abbreviations

Blast: Basic Local Alignment Search Tool
CARD: The Comprehensive Antibiotic Resistance Database
COG: Cluster of Orthologous Groups
CPR: Candidate Phyla Radiation
Diamond: Double index alignment of next-generation sequencing data
DRAM: Distilled and Refined Annotation of Metabolism
E.C.: Enzyme Commission
EggNOG: Evolutionary genealogy of genes: Non-supervised Orthologous Groups
GO: Gene Ontology
HMM: Hidden Markov Models
InterPro: Integrative Protein Signature Database
KEGG: Kyoto Encyclopedia of Genes and Genomes
KO: KEGG Orthology
MAG: Metagenome-assembled genome
MAPLE: Metabolic and Physiological potentiaL Evaluator
NCBI: National Center for Biotechnology Information
PANTHER: Protein analysis through evolutionary relationships
Pfam: Protein Families Database
PGAP: Prokaryotic Genome Annotation Pipeline
Prodigal: PROkaryotic DYnamic programming Gene-finding ALgorithm
Prokka: Prokaryotic annotation
RAM: Random-access Memory
RAST: Server for Rapid Annotations using Subsystem Technology
RefSeq: Reference Sequence Database
rRNA: Ribosomal ribonucleic acid
SAG: Single cell amplified genome
SEED: The database and infrastructure for comparative genomics
Sword: Smith–Waterman on Reduced Database
TIGRFAM: The Institute for Genomic Research's database of protein families
tRNA: Transfer ribonucleic acid
UniProt: Universal Protein Resource
