# Supplementary material for: MicrobeAnnotator: a user-friendly, comprehensive functional annotation pipeline for microbial genomes
Source: BMC Bioinformatics. 2021 Jan 6;22:11. doi: 10.1186/s12859-020-03940-5 (PMC7789693; doi:10.1186/s12859-020-03940-5)
Supplement: Supplementary file 1 — Additional file 1. Supplementary results and figures. [file 12859_2020_3940_MOESM1_ESM.pdf]

Supplementary Information

MicrobeAnnotator: a user-friendly, comprehensive microbial genome annotation pipeline

Carlos A. Ruiz-Perez[1], Roth E. Conrad[2], and Konstantinos T. Konstantinidis[1,3,4*]

[1]School of Biological Sciences, [2]Ocean Science & Engineering, School of Biological Sciences, [3]School of Civil and Environmental Engineering, [4]Center for Bioinformatics and Computational Genomics, Georgia Institute of Technology, Atlanta, Georgia, 30332

*Corresponding author: kostas@ce.gatech.edu

Table of Contents

Detailed MicrobeAnnotator output …………………………………………………………………..…. 3

MicrobeAnnotator option performance ……………………………………………………………... 4

Supplementary Figures ……………………………………………………………………………… 6

Supplementary References …………………………………………………………………………. 14

Detailed MicrobeAnnotator Output

MicrobeAnnotator creates a single output folder per run, whose structure is:

[Output Folder]/
- annotation_results/
  - [genome].annotations – Tab-separated files with annotations for each protein in the input file. The columns of this file include:
    - query_id: Identifier of query protein.
    - protein_id: Identifier of best match.
    - product: Name of best match (e.g. acyl-CoA thioesterase).
    - ko_number: KO identifier.
    - ko_product: Protein name associated with KO identifier.
    - taxonomy: Taxonomy of best match.
    - function_go: Gene Ontology identifiers associated with Function.
    - compartment_go: Gene Ontology identifiers associated with Compartment.
    - process_go: Gene Ontology identifiers associated with Process.
    - interpro: InterPro identifier.
    - pfam: Pfam identifier.
    - ec_number: E.C. number.
    - database: Database where the match was found.
  - [genome].ko – File with KO identifiers found in the genome; one per line.
- process_log/
  - log.txt - File with last step of the annotation completed.
  - structure.pickle – File with record of files created by MicrobeAnnotator
- kofamscan_results/
  - [genome].kofam – Raw output from KOfamscan
  - [genome].kofam.filt – Filtered file with best KOfamscan matches.
- refseq_results/
  - • Method indicates the search tool used (blast, diamond, or sword)
  - [genome].[method] – Blast-like tabular output.
  - [genome].[method].filt – Blast-like tabular output with best match per query.
- swissprot_results/
  - • Method indicates the search tool used (blast, diamond, or sword)
  - [genome].[method] – Blast-like tabular output.
  - [genome].[method].filt – Blast-like tabular output with best match per query.
- trembl_results/
  - • Method indicates the search tool used (blast, diamond, or sword)
  - [genome].[method] – Blast-like tabular output.
  - [genome].[method].filt – Blast-like tabular output with best match per query.
- [prefix]_barplot.pdf – Barplot with modules above 80% complete in at least one genome.
- [prefix]_heatmap.pdf – Heatmap with modules above 50% complete in at least one genome.
- [prefix]_module_completeness.tab – Tabular file with module completeness and pathway correspondence.

In summary, MicrobeAnnotator provides the user with all annotation results files to allow expert users to inspect the raw and filtered search files, improving the tool's transparency. Users can also extract additional information from the *annotation* files, such as other database identifiers like InterPro, Pfam, GO, and E.C., allowing users to compare the annotations with other databases. In this regard, we also included in the MicrobeAnnotator pipeline scripts designed to convert from one type of identifier to another. For instance, a user can take the .ko file and convert all the KO identifiers into E.C. numbers (when a match is found). The availability of other database identifiers further improves the compatibility of MicrobeAnnotator with other annotation tools and databases, allowing for easier comparisons and translation of results from one tool to another.

MicrobeAnnotator option performance

We have developed MicrobeAnnotator to be flexible and user-friendly, allowing newer users to use default options but allowing experienced users to change and select the searching methods and tools depending on their preferences. One of the options in MicrobeAnnotator allows the user to select between Blast, Diamond, or Sword as the search tool to find matches against the annotation databases. We compared all three options in terms of database building times and annotation times. Figure S2A shows the database creation time comparison between all three searching tools. Given that Sword uses raw FastA files as inputs, there is no additional time required after downloading the databases and parsing the metadata; Diamond and Blast, on the other hand, have an additional database building time of ~0.5 and ~8.4 hours, respectively. We selected ten Escherichia coli genomes (Table S1) for the annotation test and annotated them using 10 threads for each one, recording the time required for the complete process to finish. The proteins for each genome were directly downloaded from their original record in NCBI, so no protein prediction was performed for this comparison. The average time required to annotate, on average, 4,600 proteins per genome for Diamond was 26.7 minutes, compared to 221.22 minutes (~3.6 h) for Sword and more than 36 hours for Blast (Figure S2B). Another important feature of MicrobeAnnotator is its iterative pipeline that only uses the subset of unannotated proteins in the previous step to reduce the search space against larger databases; we have implemented an option if the user wants to annotate all proteins against all databases. This mode is called the *full* mode of MicrobeAnnotator. We estimated the time required to perform the full annotation, i.e., search all proteins with KOfamscan, and against Swissprot, RefSeq, and trEMBL using Diamond, the fastest method available. The difference between the standard and full annotation versions using diamond was ~10 minutes on average for a typical *E. coli* genome.

Besides speed, annotation consistency is also an important factor of functional annotation; to evaluate consistency, we used three different metrics. The first metric compared the annotations based on KO identifiers recovered for all three methods, the second estimated the number of annotated, hypothetical, and unannotated proteins, and the third compared the protein annotations themselves. The first metric (Figure S1), showed that all three methods recover the same or almost the same module completeness in all genomes used, with a small difference in completeness levels in the ETEC colonization factors - pathogenicity signature module. The second metric compared the type and number of annotations, and we defined annotation types as previously described [1]. We considered a protein as annotated when it had at least one annotation from any database that did not contain 'hypothetical,' 'uncharacterized,' 'domain of unknown function,' or 'protein of unknown function;' a hypothetical protein, on the other hand, had a match in any database but contained any of the terms above. Finally, a protein was classified as unannotated when it had no match against any of the databases (identified as "No match found" in MicrobeAnnotator results). Figure S2C shows the comparisons on the percentage of proteins annotated using all three search tool methods. As expected, most proteins from *E. coli* genomes are classified as annotated using any method, while a small minority remains unannotated. There were no statistically significant differences between the median percentage of *annotated* and *hypothetical* proteins among the methods used (Kruskal-Wallis, p>0,05). However, there were significant differences between the median percentage of proteins that remained unannotated (Kruskal-Wallis, H=22,2, p<0,01), with Sword having the lowest value, followed by Blast. Finally, the third comparison method involved estimating the similarity between the text-based annotations obtained using each method. Given that it is challenging to manually compare the annotation text of two sets of proteins and considering that annotation descriptions between proteins can vary even if they are the same protein, we calculated the cosine similarity between a pair of annotation texts. The cosine

similarity has been widely used to measure how similar text documents or strings are to one another [2]. Briefly, the text associated with the annotations obtained was transformed into a vector of unique words that is compared with another; if two annotations are identical, they have a similarity of 1. Otherwise, depending on the number of words they share, the similarity ranges between 0 and 1. Figure S2D shows the cosine similarities between the annotations obtained using Blast and Sword for all proteins in each *E. coli* genome tested. Most proteins have identical or almost identical text-based annotations suggesting both methods find the same best match most of the time. There are some outliers in all genomes that, upon closer inspection, show small differences in the annotations, such as additional gene names or instances of "putative" vs. "probable." On the other hand, comparisons between Blast vs. Diamond, or Sword vs. Diamond (Figure S3), showed that several of the outliers are proteins that were not annotated using Diamond. This result further supports our previous findings that suggest Blast and Sword are more sensitive methods than Diamond. Therefore, all our comparative methods with other tools use Sword as the default method given that it has a similar sensitivity to Blast but is much faster. In addition, we recommend the use of Sword for detailed annotations, while Diamond can be used for quick annotation and visualization purposes.

One key feature of MicrobeAnnotator is its capacity to simultaneously process multiple genomes and combine their annotation information in a single plot. While other tools can accept multiple genomes (jobs), they are processed separately, reporting the results individually. MicrobeAnnotator simultaneously processes genomes across threads to significantly speed up the analysis of batch genome collections, reporting each genome's annotations while also compiling a matrix with KO identifiers and report KEGG module completeness in two plots for efficient cross-genome comparisons (Figure S1). Estimates of run times evaluated with 50 *E. coli* genomes and the light version of MicrobeAnnotator showed that the use of multiple computation threads speeds up the annotation process overall. The speedup gains obtained by this multiprocessing execution fall between 90-95% parallelization efficiency as described by Amdahl's Law (Figure S4), where it grows sub-linearly with each added computation thread. Nonetheless, the speedup depends on the exact genomes used and the number of initial proteins found in the smaller database used by KOfamscan.

In summary, considering the tradeoffs between annotation times and consistency, Diamond appeared to be a feasible and fast option for rapid annotation of genomes. At the same time, Sword emerged as an alternative for more sensitive annotation without the limitations in computational time that Blast showed in our tests. By taking advantage of the multiprocessing options within MicrobeAnnotator, users can use the full computing power available to parallelize the annotation of multiple genomes more efficiently while retaining the ability to obtain individual results and summaries using all genomes in the initial input.

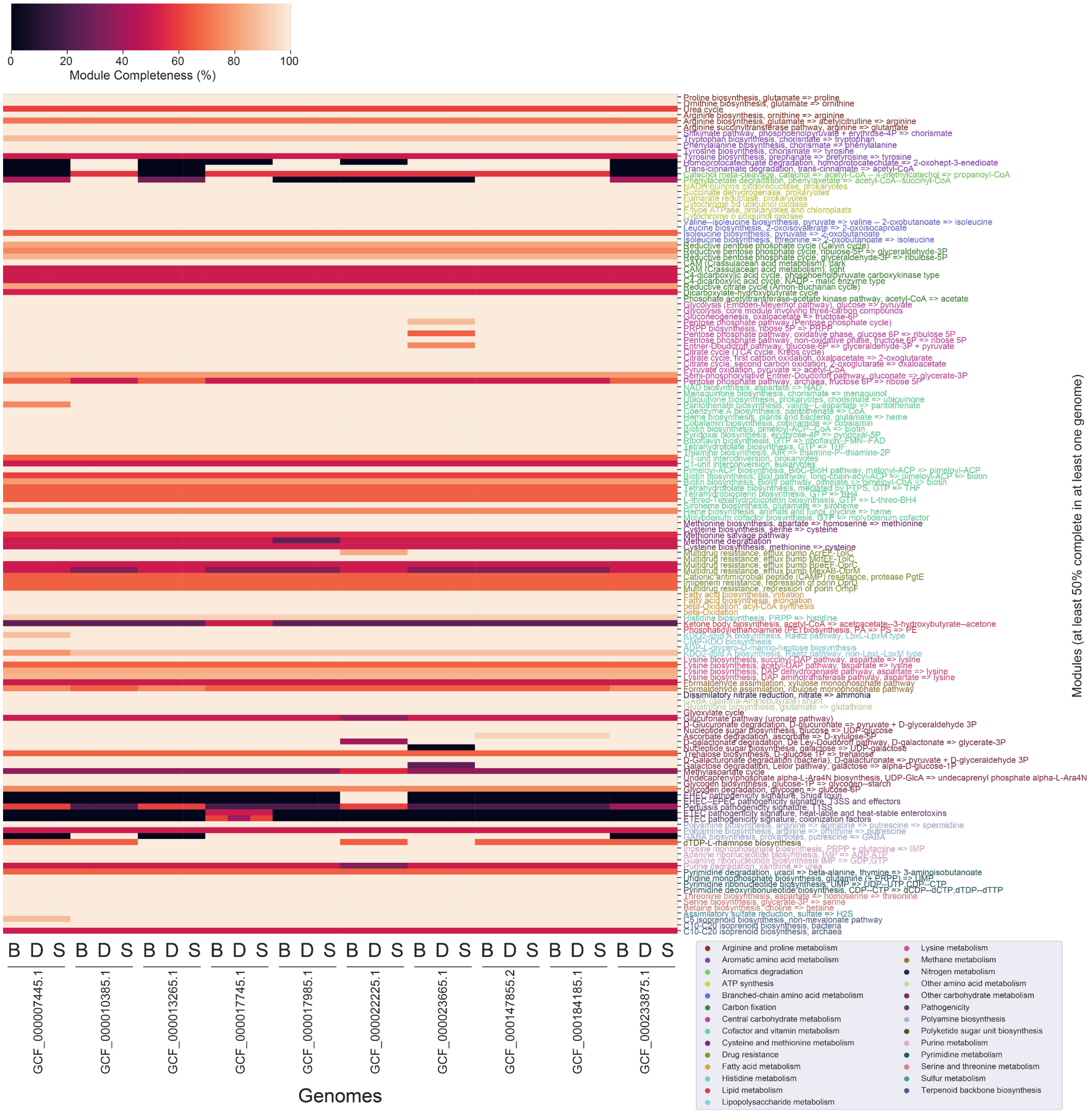

Figure S1: Annotation comparison between search tool options. The heatmap shows module completeness (y-axis) for 10 *E. coli* genomes (x-axis) annotated using the three different search methods available in MicrobeAnnotator (Blast, Diamond, and Sword). Note the completeness level of all modules recovered is identical or almost identical for all genomes. The different colors of the modules indicate which pathway they belong to (lower right box).

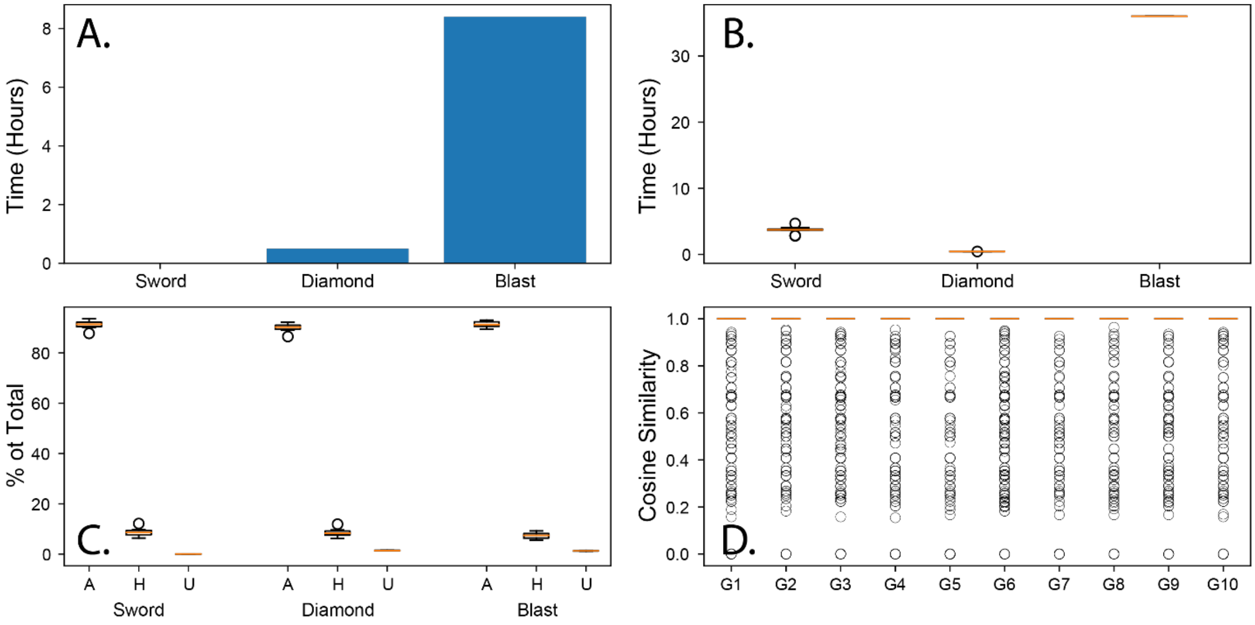

Figure S2: Performance and consistency comparison among search tool options. The different search tool options available in MicrobeAnnotator were compared in terms of (**A.**) Additional time (in hours) required to build the search databases, (**B.**) the time required to annotate a typical bacterial genome (~4600 proteins), and (**C.**) the percentage of proteins classified as *annotated* (*A*), *hypothetical* (*H*), and *unannotated* (*U*). Finally, the consistency of annotations between Blast and Sword estimated using cosine similarities showed that most annotations are identical for the ten genomes tested (G1-G10, Table S1; **D.**).

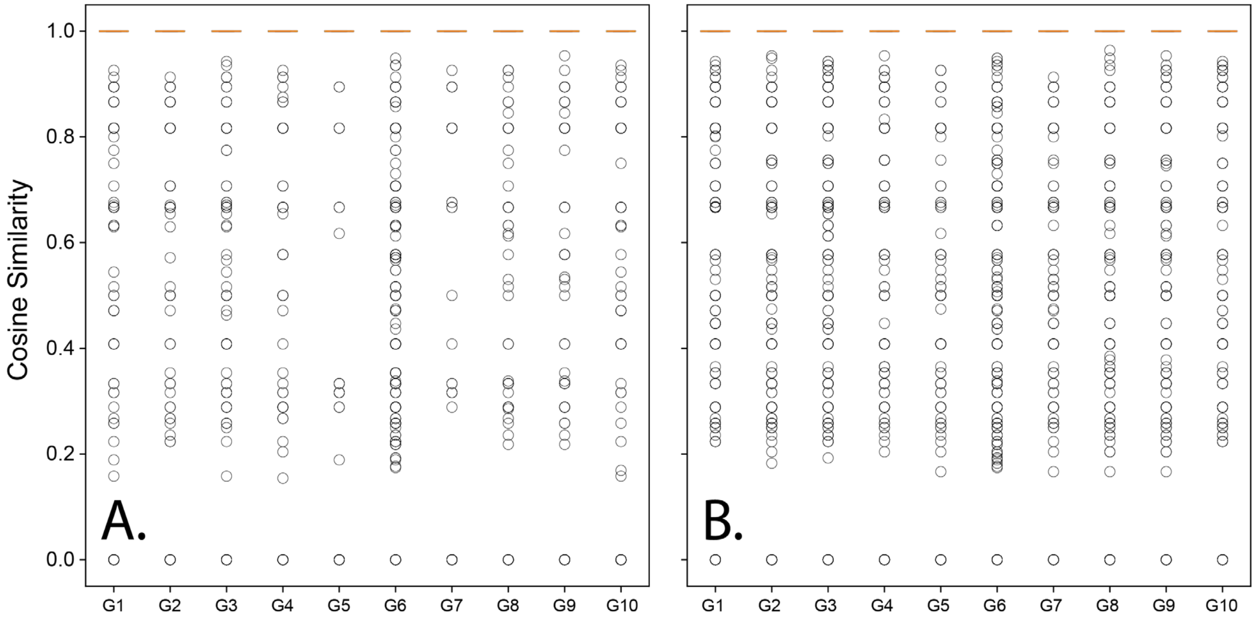

Figure S3: MicrobeAnnotator annotation similarity among search tools. The similarity of text-based annotations obtained using MicrobeAnnotator and different search tool options indicated that Blast vs. Sword (**A.**) and Sword vs. Diamond (**B.**) annotations are mostly identical, revealing that the tools find the same best matches overall.

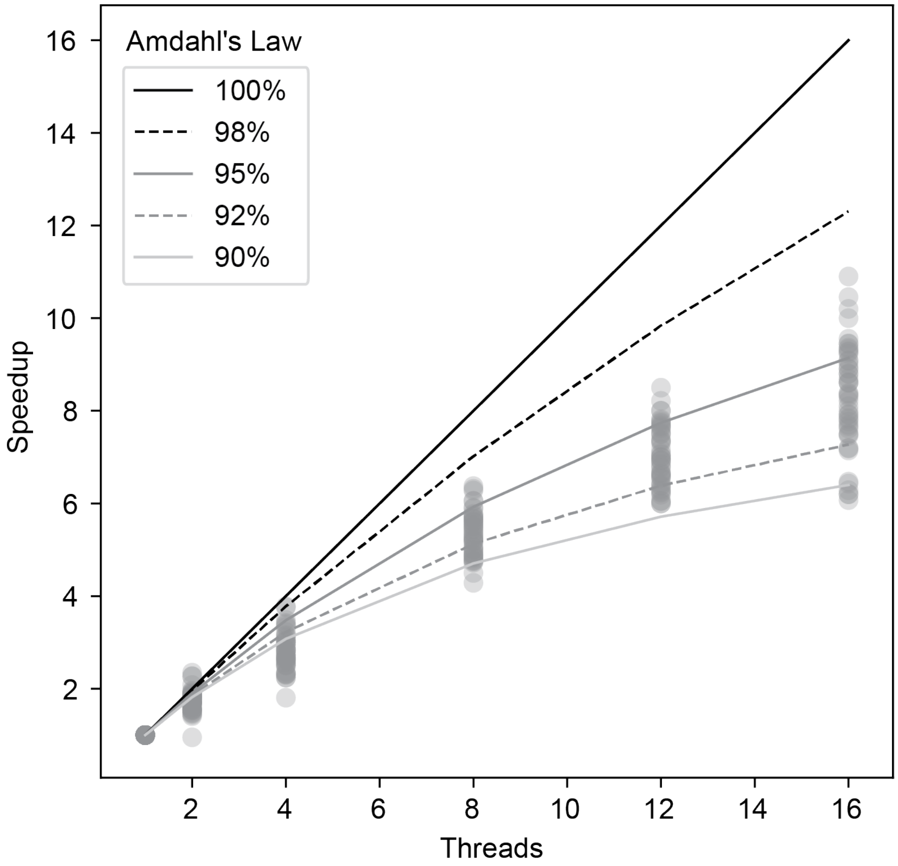

Figure S4: MicrobeAnnotator multithreading computation speedup. The speedup was compared using the *light* mode in MicrobeAnnotator. Times were compared with the base computation using one thread. Note that increasing the number of threads results in a speedup comparable to ~95% parallelization according to Amdahl's law.

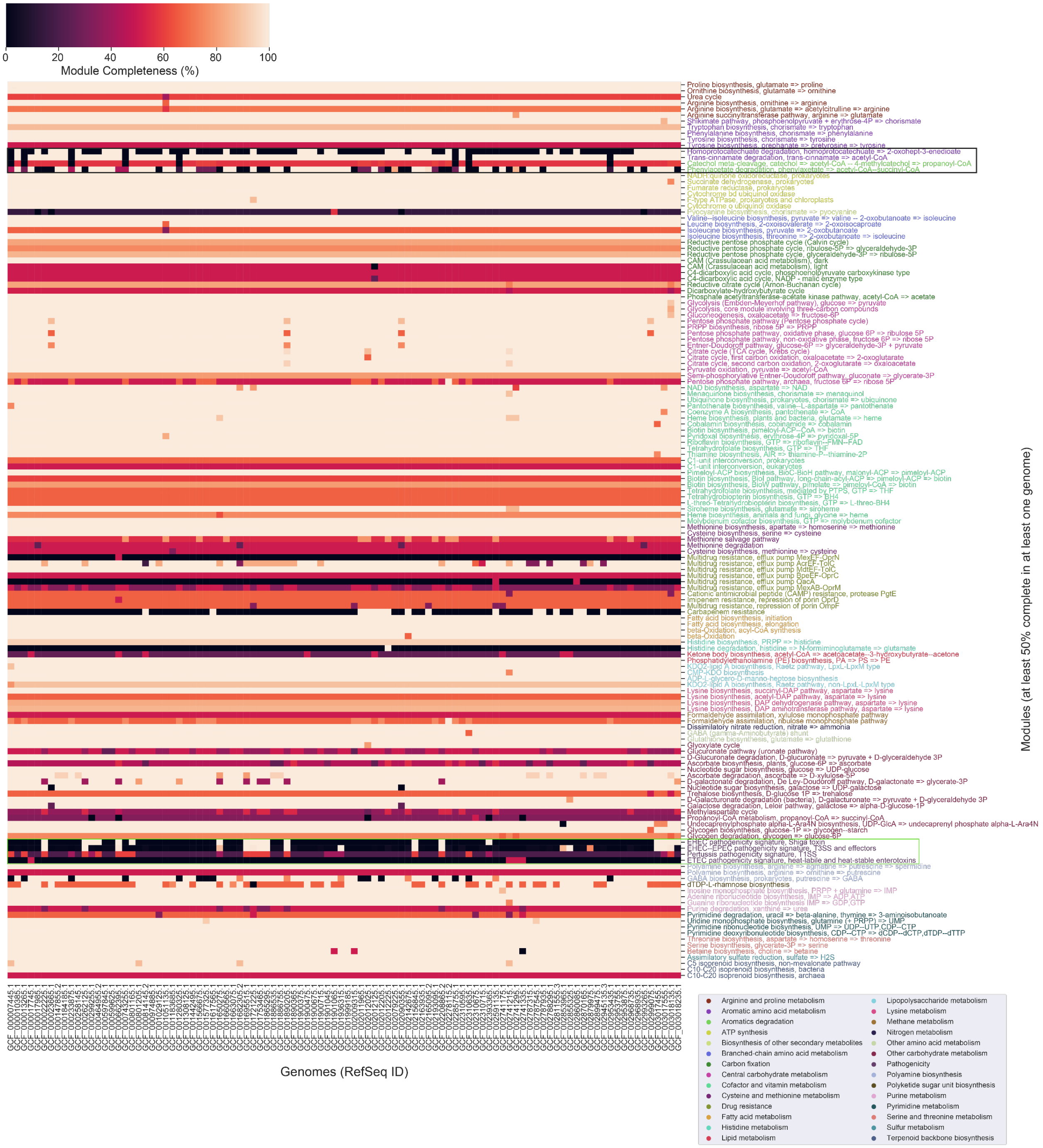

Figure S5: Module completeness heatmap of 100 *E. coli* genomes. Despite the overall similarity in central carbon, vitamin, and amino acid metabolism expected among *E. coli* genomes, even small differences in the metabolic potential can be easily identified using the heatmap. For example, note the differences in module completeness in pathogenicity-related modules (green box) and aromatic compounds metabolism (black box). These small differences can serve as differentiating factors even between close relatives.

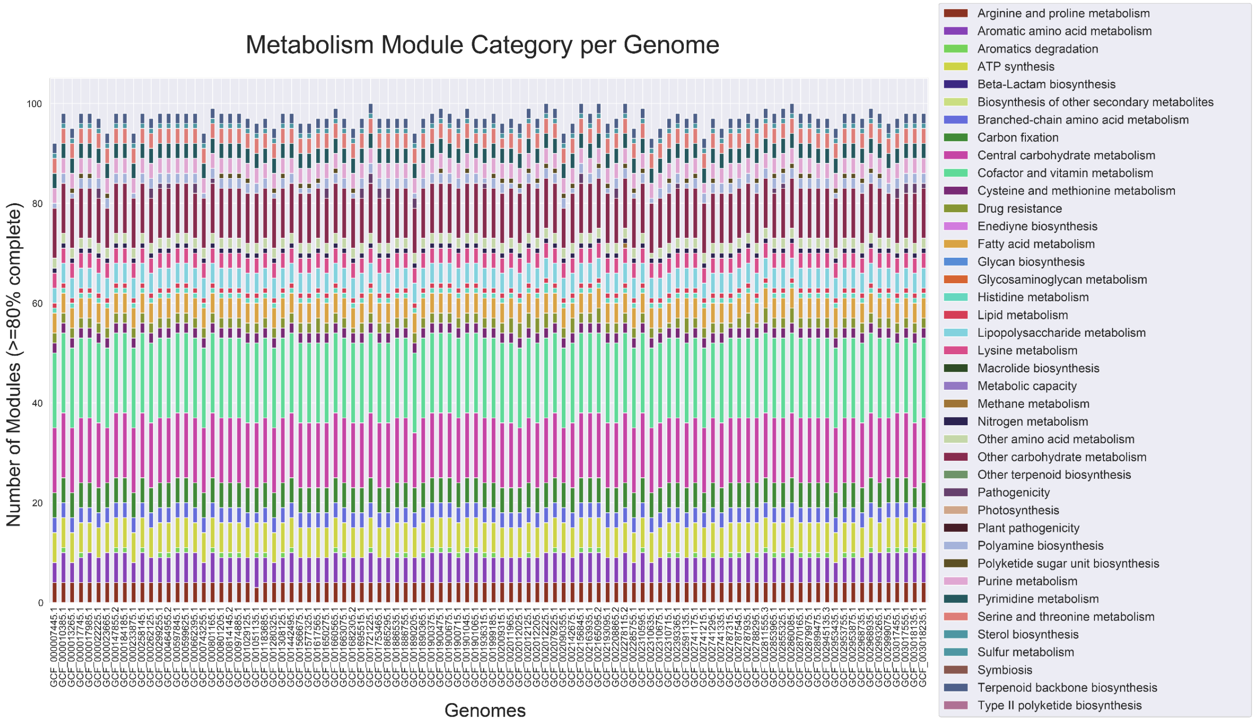

Figure S6: Barplots of modules with completeness above 80% grouped by the category (pathway). Note the high similarity in metabolic pathways exhibited by all *E. coli* genomes analyzed. Such plots can easily reveal differences in pathway presence/absence among the genomes being compared. In the specific case of *E. coli* genomes, not many differences are evident due to overall similar metabolic potential encoded in most genomes.

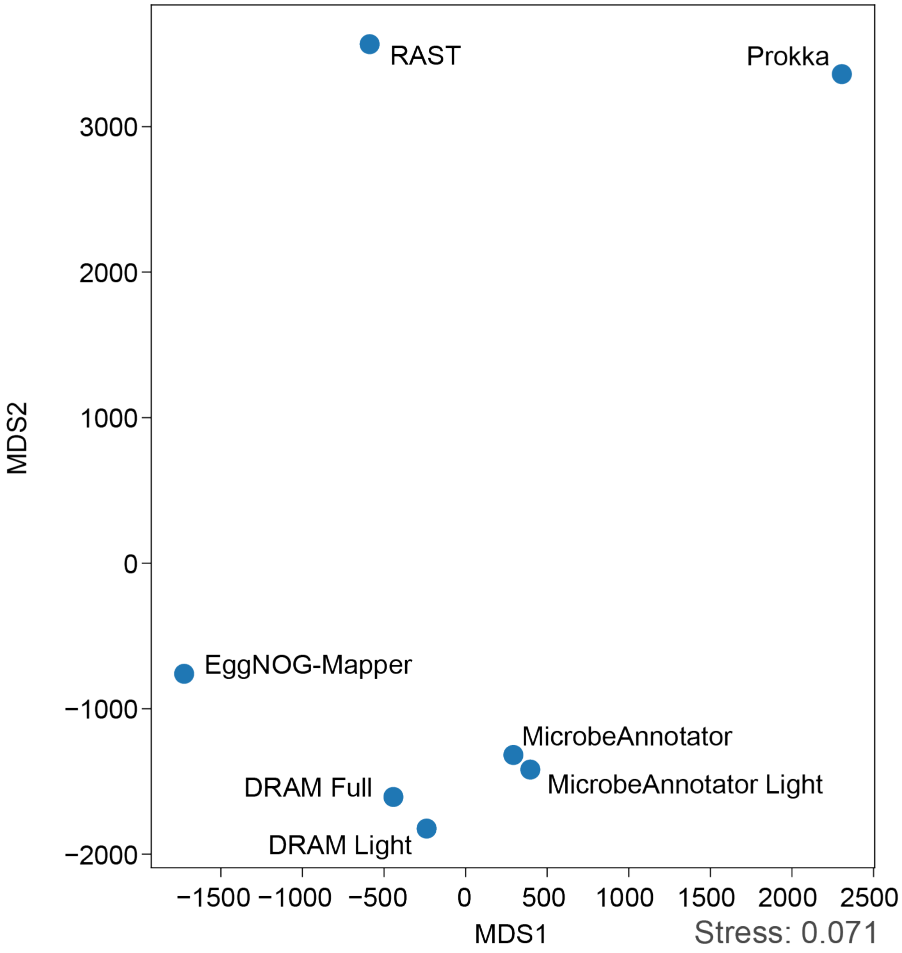

Figure S7: Multi-dimensional scaling ordination of annotation matrix distances. The distance between annotation summary matrices for *E. coli* genomes showed that the two modes of MicrobeAnnotator recover similar KO-based summaries similar to those obtained using DRAM. Prokka and RAST tend to recover different modules highlighting the advantage of using multiple annotation tools and incorporating their results into MicrobeAnnotator.

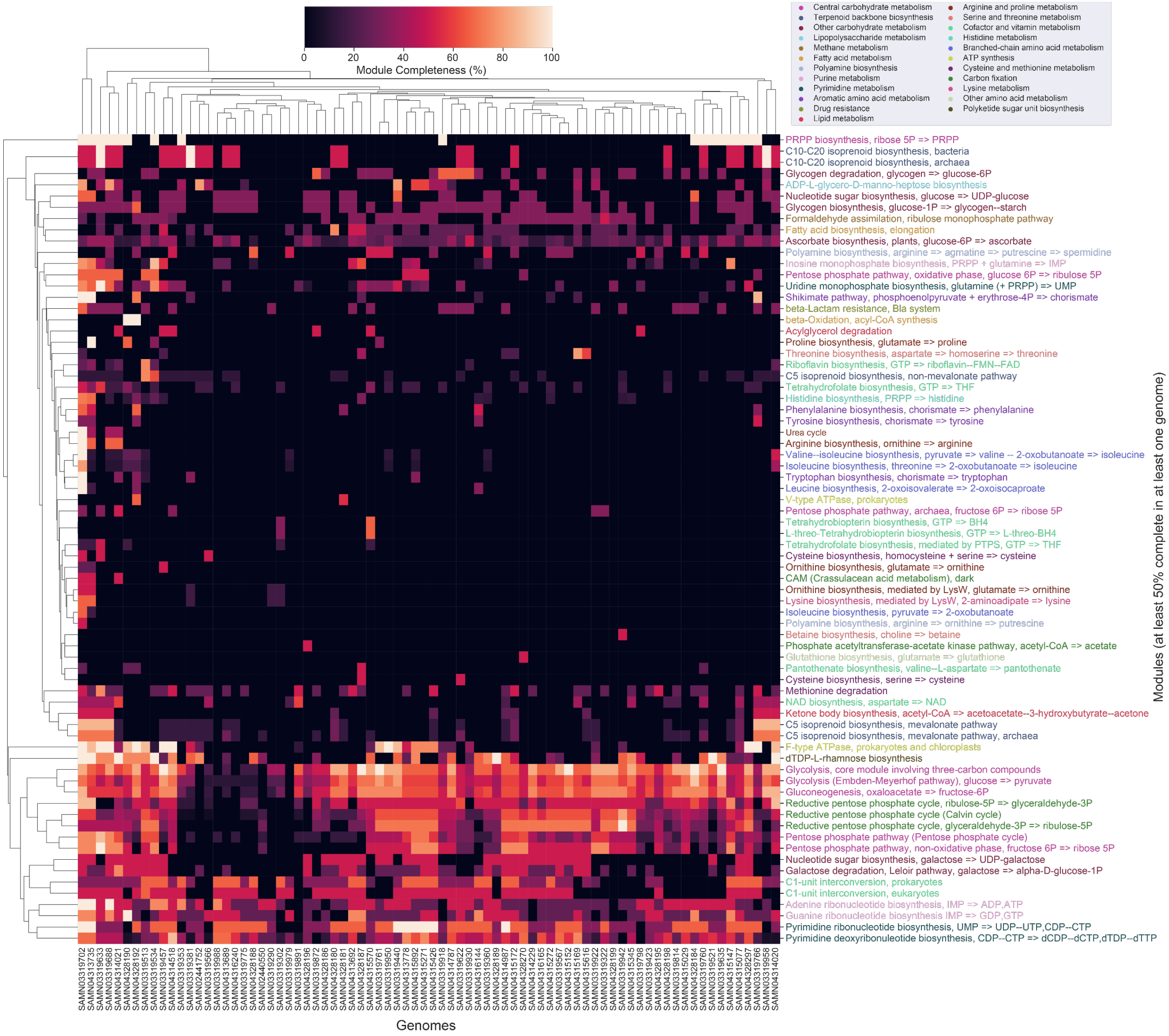

Figure S8: Heatmap of module completeness of 78 Candidate Phyla Radiation MAGs and SAGs. The heatmap shows some conservation in primary metabolism, including glycolysis, pentose phosphate cycle, and biosynthesis of nucleotides among the CPR genomes. Some genomes also encode the potential for Glycogen degradation/biosynthesis, isoprenoid biosynthesis, and formaldehyde assimilation. The most striking pattern is the lack of many modules or the low completeness of those recovered in most genomes, reflecting a possible limited metabolic versatility or a high degree of MAG and SAG incompleteness.

References

1. Shaffer M, Borton MA, McGivern BB, Zayed AA, La Rosa SL, Solden LM, Liu P, Narrowe AB, Rodriguez-Ramos J, Bolduc B *et al*: **DRAM for distilling microbial metabolism to automate the curation of microbiome function**. *Nucleic Acids Res* 2020, **48**(16):8883-8900.
2. Han J, Kamber M, Pei J: **2 - Getting to Know Your Data**. In: *Data Mining (Third Edition)*. Edited by Han J, Kamber M, Pei J. Boston: Morgan Kaufmann; 2012: 39-82.
